# Inflammation-mediated effects of diabetes mellitus on male fertility: a systematic review and meta-analysis

**DOI:** 10.3389/fendo.2025.1600565

**Published:** 2025-11-17

**Authors:** Yanan Hao, Zheng Yang, Yanni Feng, Yong Zhao, Yonglin Ren

**Affiliations:** 1 The Institute of Brain Science and Brain-inspired Research, Shandong First Medical University and Shandong Academy of Medical Sciences, Jinan, China; 2 College of Environmental and Life Sciences, Murdoch University, Perth, WA, Australia; 3 College of Pharmacy, Shandong First Medical University, Jinan, China; 4 College of Life Sciences, Qingdao Agricultural University, Qingdao, China; 5 Chinese Academy of Agricultural Sciences, Beijing, China

**Keywords:** type 1 diabetes, type 2 diabetes, inflammation, male infertility, meta-analysis

## Abstract

**Background:**

Diabetes mellitus (DM) has emerged as a rapidly growing global health problem, imposing substantial socioeconomic burdens and multidimensional health consequences, including adverse effects on male fertility. Although accumulating evidence suggests associations between DM and male reproductive dysfunction, comprehensive mechanistic insights, particularly through inflammatory pathways, remain inadequately elucidated.

**Method:**

We conducted a systematic literature search on Web of Science, Embase, and PubMed databases (1972–2022) to investigate DM-related male infertility through meta-analysis. Following PRISMA guidelines, eight of 168 studies on type 1 diabetes (T1D) and nine of 185 studies on type 2 diabetes (T2D) were included to screen the relationship between diabetes and male infertility. In addition, 10 of 840 inflammation-related studies (1961–2022) underwent rigorous selection for mechanistic exploration. Meta-analysis was conducted to evaluate the summary relative risk (RR) and 95% confidence intervals (CIs) across the combined studies.

**Results:**

Meta-analysis demonstrated a significant impairment of male fertility in diabetic populations. Subgroup analyses revealed that T2D is more likely to cause male infertility compared to T1D. Despite low between-study heterogeneity, inflammation biomarkers (e.g., TNF-α) were implicated in diabetes-induced male infertility. Transcriptomic analyses further identified enriched inflammatory pathways and altered expression of fertility-related genes.

**Conclusion:**

Current evidence indicates that diabetes adversely affects male fertility through inflammatory pathways.

## 1 Introduction

Diabetes mellitus (DM) is a cluster of chronic disorders characterized by persistent hyperglycemia resulting from impaired insulin secretion (type 1 diabetes [T1D]) and/or insulin resistance (type 2 diabetes [T2D]) (1). According to the International Diabetes Federation (IDF), in 2021, DM affected over 1.2 million pediatric T1D cases, and 541 million adults were at high risk of T2D worldwide. DM has emerged as a critical global issue, with significant social, health, and economic consequences (2). T1D can develop at any age, but it is most prevalent among children and adolescents, who produce very little or no insulin. Conversely, T2D is more common in adults and accounts for approximately 90% of all diabetes cases, characterized by impaired insulin utilization (1, 71). Emerging evidence links DM to male reproductive dysfunction, particularly through sperm abnormalities (3, 32). Mechanistic studies implicate multiple pathways, including proinflammatory responses (4–6), oxidative stress (7, 8), hormone dysregulation (luteinizing hormone [LH], follicle-stimulating hormone [FSH], testosterone [T]) (9–12), increased glucose (13), and sperm DNA fragmentation (14, 15). However, significant heterogeneity across studies arises from differences in sample characteristics (age, disease duration, sizes), methodological variability (biomarker selection), and conflicting outcome measures, which hinders the conclusive synthesis of DM-mediated male infertility mechanisms.

Male infertility is defined as the inability to conceive following 1 year of twice-weekly unprotected intercourse (16). An increasing body of evidence indicates that male infertility may be a harbinger of future adverse health outcomes (17). Numerous factors contribute to male infertility, including genetic abnormalities (18, 19) and lifestyle risk factors (e.g., environment, nutrients, smoking, stress, and endocrine disruptors) (20). These factors may lead to immunologic disorders and sperm dysfunction (21), testicular disorders (16), and oxidative stress (22). Unhealthy lifestyles, including high-fat or high-sugar diets, are often accompanied by diabetes. Studies suggest that diabetes negatively impacts male fertility both directly and indirectly, affecting spermatogenesis, penile erection, and ejaculation (23–26).

As mentioned above, numerous studies have investigated the relationship between diabetes and male fertility. Despite growing recognition of DM-associated male infertility, current evidence remains fragmented due to inconsistent experimental designs and limited mechanistic integration. Meta-analysis, a robust statistical method for synthesizing heterogeneous datasets (27, 28), offers a solution by quantifying pooled effect sizes and identifying modulatory factors. In this study, we employed a systematic meta-analysis combined with bioinformatics validation to quantify the magnitude of DM-induced male fertility impairment across T1D/T2D subtypes, elucidate inflammation-centric mechanisms through pathway enrichment analysis, and establish evidence-based priorities for future therapeutic interventions.

## 2 Methods

Meta-analyses were carried out in accordance with the published guidelines of Meta-Analysis of Observational Studies in Epidemiology and the Preferred Reporting Items for Systematic Reviews and Meta-Analyses.

**Figure 1:**
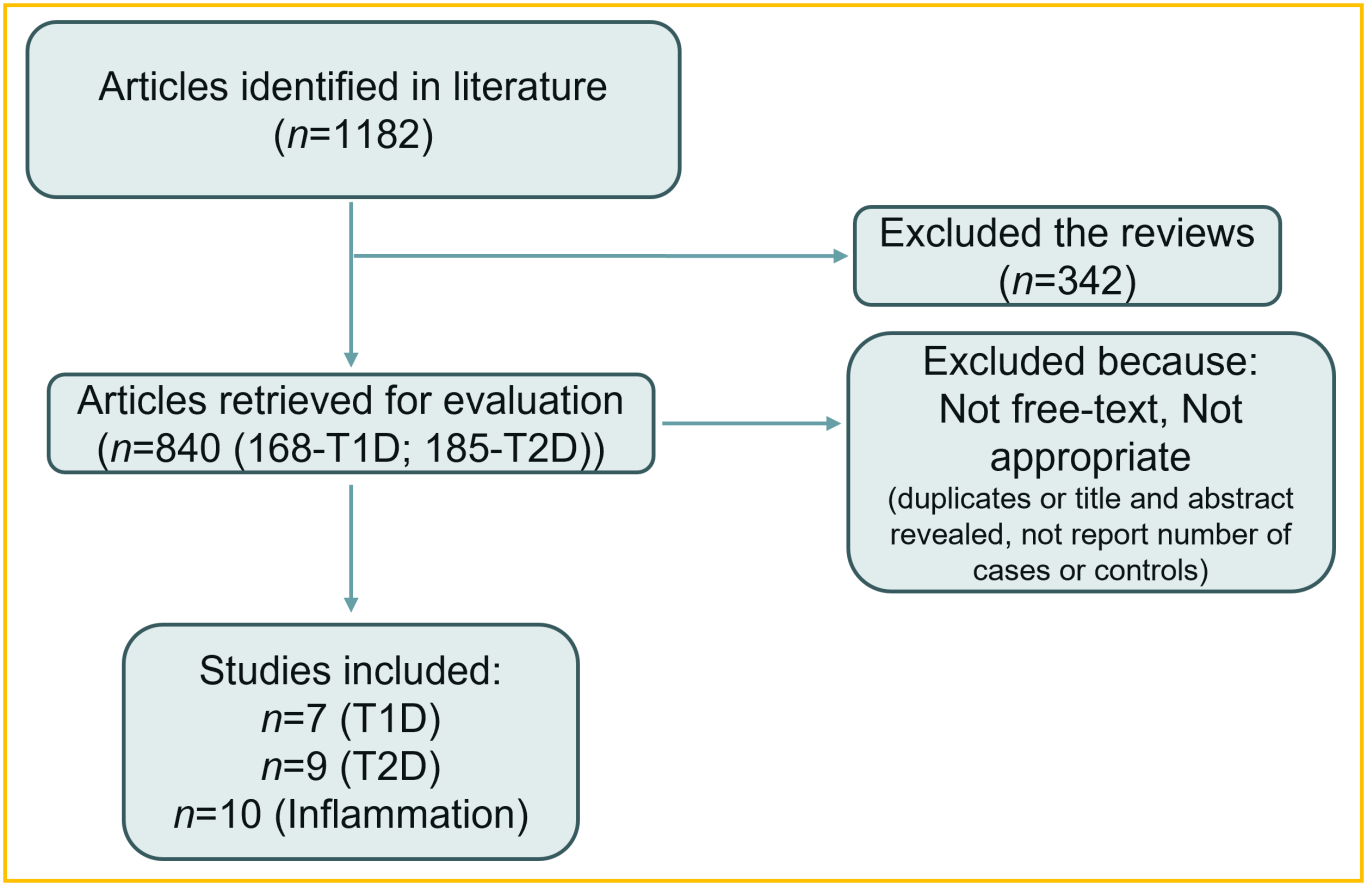
Flow diagram of the papers selected for the meta-analysis.


We conducted a meta-analysis to explore the relationship between diabetes and male infertility. The literature included in this study was retrieved from PubMed, Embase, and Web of Science. The search terms diabetes or diabetes mellitus, male infertility or sterility, inflammation or immune response or cytokines or chemokines were used to search titles, keywords, and abstracts using the fuzzy search option. The studies included were published between 1961 and 2022. A flowchart of the selected papers is presented in **Figure 1**. Given the limited number of studies, statistical tests for publication bias were not performed. Heterogeneity was expressed using the *I*-squared test, where a higher *I*-squared indicates greater heterogeneity. The *Z*-test was used to represent the cumulative probability of the total effect. The formula for the *Z*-test is as follows (29):

Z=(x−μ)/(σ / √n)

Where: *x* is the sample mean; *μ* is the overall mean; ó is the overall standard deviation; and *n* is the sample size. *Z*-value represents the different probability (*Z* = ~ − ∞: 0%; *Z* = ~ 0: 50%; *Z* = ~ + ∞: 100%).

A total of 1,182 papers were initially retrieved. The titles and abstracts were screened, and studies containing information on diabetes mellitus and male fertility were selected. Among these, 840 experimental studies were first screened to ensure specific data support for the subsequent meta-analysis. Of these, 168 papers were related to type 1 diabetes, and 185 papers were related to type 2 diabetes. The publications were then assessed and selected if they met all the following criteria:

- The aim of the study was to evaluate the influence of diabetes on male fertility.
- The full text of the study was available.
- The study was a primary research paper, not a review article.
- The study reported the number of individuals and included at least two groups (control and treatment).
- Duplicate articles were excluded.

After the screening, 27 papers met the inclusion criteria. The selected articles were analyzed using RevMan v5.4 (Cochrane, London, UK). These studies were then divided into T1D (eight papers), T2D (nine papers), and inflammation (10 papers) groups. In this study, the fixed-effect model was applied as follows (30):


$$Y_{i}=\Theta +\epsilon_{i}$$

Where: *Y_i_* is the observed effect in the study; *Θ* is the true effect in the study; and *ϵ_i_* is the difference between the true effect and the observed effect.

After the analysis, the value of the *I*-square was used for quantifying the heterogeneity, and the value of the *Z*-test was used for comparing the cumulative probability of the occurrence of the total effect amount.

**Figure 2:**
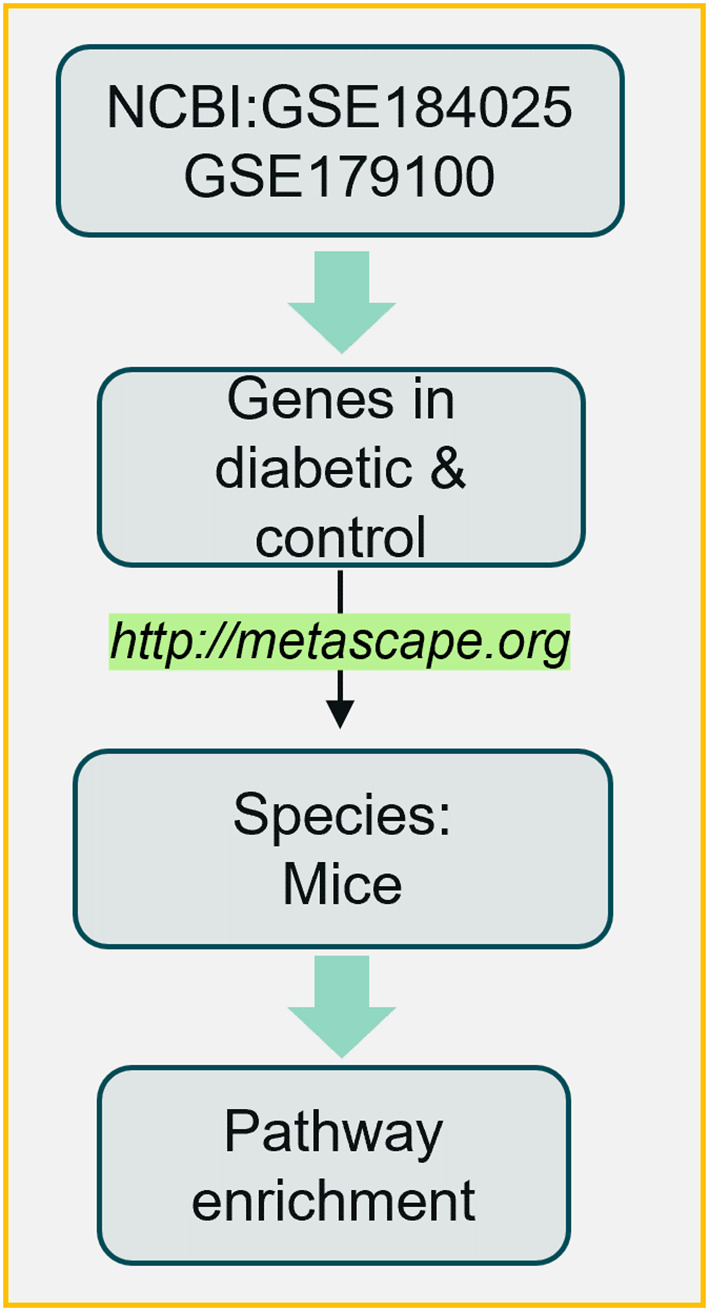
Bioinformatics pipeline (https://metascape.org/gp/index.html#/main/step1).

Gene expression levels in RNA sequencing data were estimated using the fragments per kilobase of transcript per million mapped reads (FPKM) method. To screen differentially expressed genes (DEGs), the criteria were set at *p* < 0.05 and fold change > 2. Subsequently, enrichment analysis was performed on the identified DEGs to characterize their functional roles. The number of DEGs included in each functional term was counted, and the significance of enrichment (represented by *p*-value) was calculated using the hypergeometric distribution test. A smaller *p*-value indicates a higher likelihood that the DEGs are enriched in the corresponding functional term, suggesting a nonrandom association between the DEGs and the biological process/pathway. In this study, two RNA-seq databases (National Center for Biotechnology Information [NCBI]: GSE184025 and GSE179100) from the testes of diabetic male mice were further analyzed using Metascape to obtain the enriched pathways. Briefly, the gene list was first uploaded to the Metascape website, the species (human and mice) were selected, and expression analysis was then performed. The enriched pathways were subsequently obtained, as shown in **Figure 2**.

Correlation analysis between inflammation, male fertility parameters, and glucose levels was performed using the Pearson correlation coefficient by SPSS (IBM, V.20). The data were extracted from published papers (9, 12, 13). The formula was as follows (31):


$$P_{\left(X,Y\right)}=\text{cov}(X,Y)/(\sigma_{X},\sigma_{Y})$$

Where: *P*_(_*_X, Y_*_)_ is the Pearson correlation coefficient of two continuous variables (*X*, *Y*); cov(*X*, *Y*) is the covariance between them; and *σ_X_*, *σ_Y_* are the standard deviations, respectively.

## 3 Results

### 3.1 Diabetes causes male infertility

**Figure 3:**
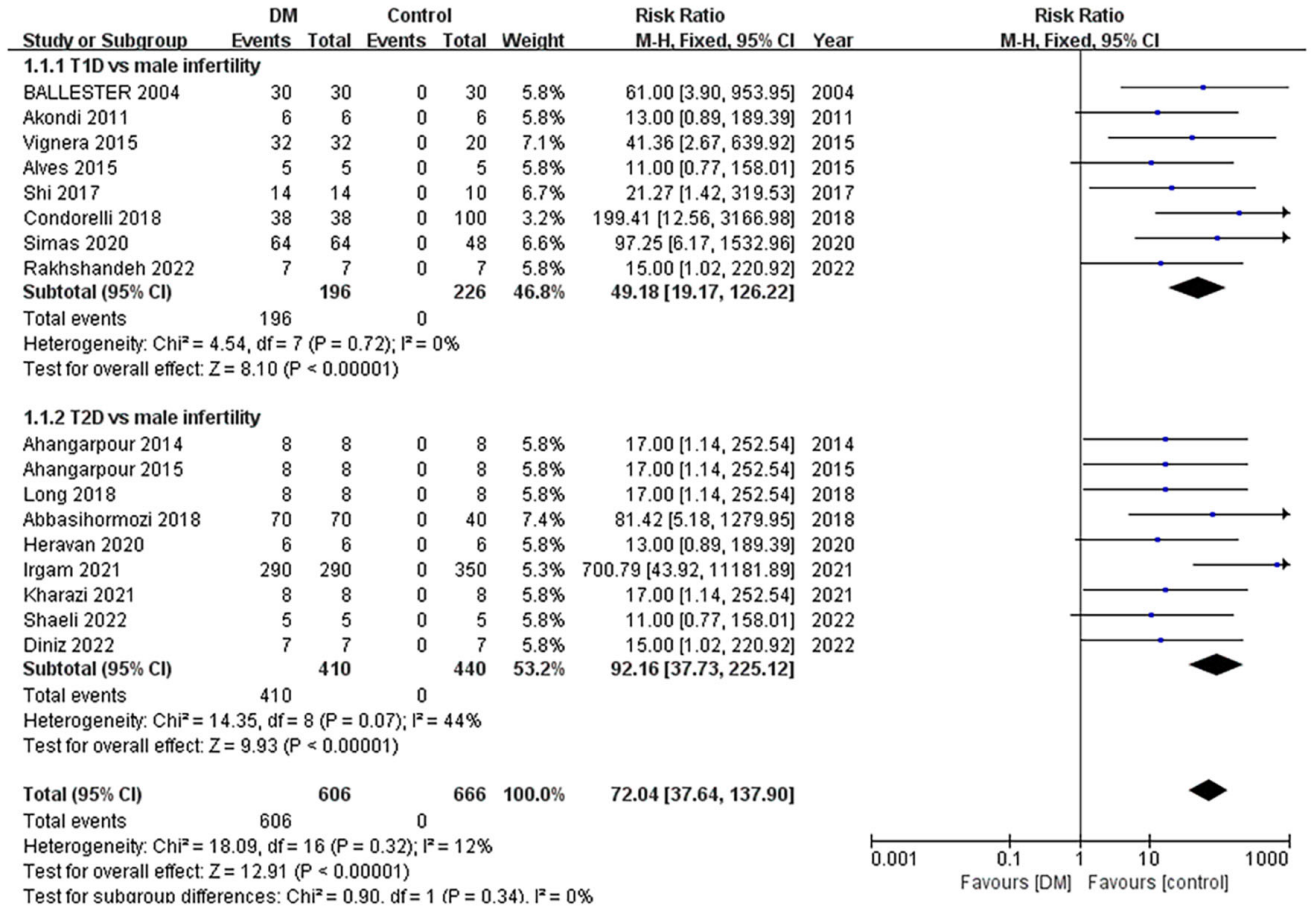
Meta-analysis of the association between diabetes and male infertility. (T1D, type 1 diabetes; T2D, type 2 diabetes).

To objectively evaluate the association between DM and male infertility, we conducted a meta-analysis approach utilizing the fixed-effect model. The included studies are presented in **Tables 1**, **2**. Eight papers analyzed T1D vs. male infertility, involving 196 individuals in the diabetic group and 226 control individuals. Nine articles examined T2D vs. male infertility, including 410 diabetic individuals and 440 controls. According to the forest plot (**Figure 3**), there were more cases of male infertility in the diabetes group than in the control group. The total *Z*-test value (*Z* = 12.91; *p <* 0.00001) indicates that diabetes significantly reduces male fertility, and the heterogeneity was not significant (RR = 72.04; 95% confidence interval [CI] = 37.64, 137.90; *I*-square = 12%). Based on the *Z*-test of the subgroup meta-analysis, T2D (RR = 92.16; 95% CI = 37.73, 225.12; *Z* = 9.93; *p <* 0.00001) showed a higher probability of causing male infertility than T1D (RR = 49.18; 95% CI = 19.17, 126.22; *Z* = 8.10; *p <* 0.00001). Heterogeneity remained nonsignificant in T1D studies (*I*-square = 0%) and moderate in T2D studies (*I*-square = 44%), suggesting greater consistency in T1D-related infertility outcomes (**Supplementary Figure S1**).

**Table 1:** Summary of the information on male infertility in T1D.

References	Country	Model types	Sample size	Male infertility in T1D
Condorelli et al. (3)	Italy	T1D patients	138	Increased
Simas et al. (15)	Brazil	T1D patients	112	Increased
Vignera et al. (32)	Italy	T1D patients	52	Increased
Shi et al. (33)	China	T1D male mice	24	Increased
Alves et al. (34)	Portugal	T1D patients	10	Increased
Rakhshandeh et al. (11)	Iran	T1D patients	14	Increased
Ballester et al. (35)	Spain	T1D male mice	60	Increased
Akondi et al. (36)	India	T1D rats	12	Increased

*T1D*, type 1 diabetes.

**Table 2:** Summary of information on male infertility in T2D.

References	Countries	Model species	Sample size	Male infertility in T2D
Diniz et al. (37)	Portugal	T2D rats	14	Increased
Long et al. (38)	China	T2D rats	16	Increased
Ahangarpour et al. (39)	Iran	T2D male mice	16	Increased
Ahangarpour et al. (40)	Iran	T2D male mice	16	Increased
Abbasihormozi et al. (41)	Iran	T2D patients	110	Increased
Kharazi et al. (10)	Iran	T2D rats	16	Increased
Rahimiyan-Heravan et al. (42)	Iran	T2D male rats	12	Increased
Al-Shaeli et al. (43)	Iraq	T2D male mice	10	Increased
Irgam et al. (44)	India	T2D patients	640	Increased

*T2D*, type 2 diabetes.

### 3.2 Inflammation assumes crucial roles in diabetes-induced male infertility

**Figure 4:**
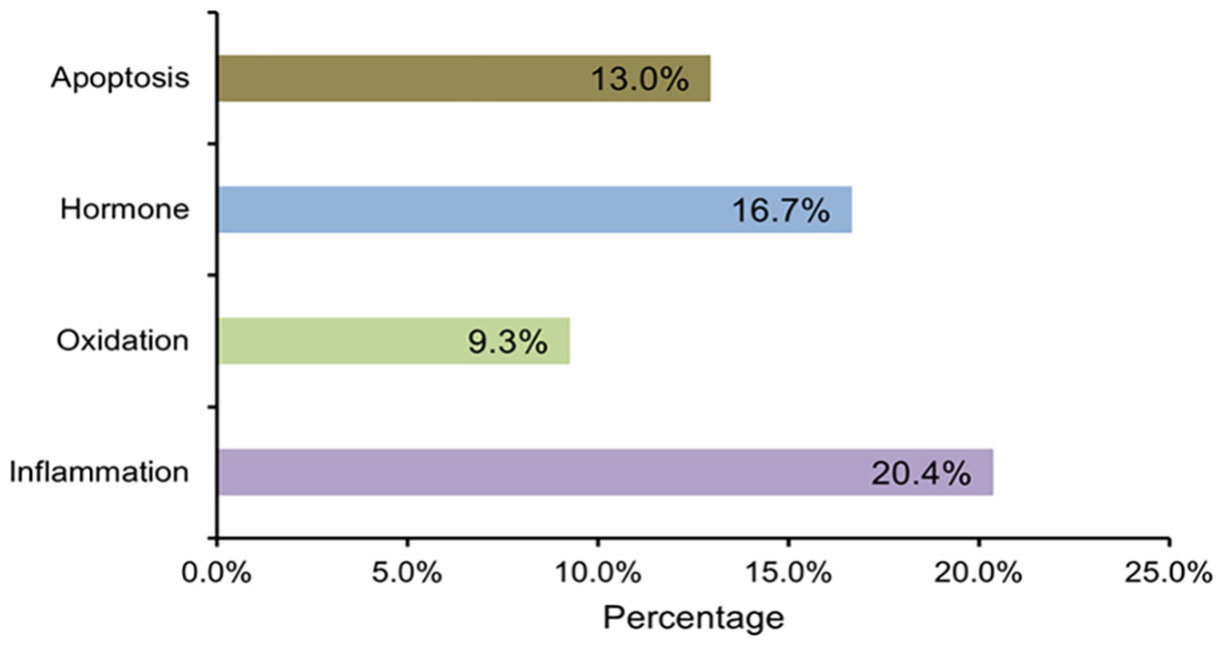
Factors contributing to male infertility caused by diabetes.

To identify the mechanisms of diabetes-induced male infertility, we summarized and calculated the frequency of the contributing factors. These included inflammation, oxidation, hormonal (e.g., FSH, testosterone), and apoptosis pathways involved in male infertility. Among them, inflammation was the most frequent factor, accounting for 20.4% (**Figure 4**). Given its predominance among the identified factors, inflammation was selected for in-depth meta-analytical validation. Eleven studies were included in the subsequent analysis (**Table 3**).

**Table 3:** Summary of the studies related to inflammation in DM-caused male infertility.

References	Country	Model types	Sample size	Inflammatory reaction in DM-male infertility
Condorelli et al. (3)	Italy	DM	193	Increased
Skurikhin et al. (45)	Russia	T1D	20	Increased
Maresch et al. (46)	Germany	T1D	20	Increased
Rakhshandeh et al. (11)	Iran	T1D	14	Increased
Heeba and Hamza (47)	Egypt	T1D	16	Increased
Han et al. (48)	China	T1D	140	Increased
Khalil et al. (49)	Malaysia	T2D	12	Increased
Nna et al. (7)	Malaysia	T1D	16	Increased
Jiang et al. (5)	Germany	DM	20	Increased
Bakhshwin et al. (4)	Saudi Arabia	T2D	20	Increased

*DM*, diabetes.

**Figure 5:**
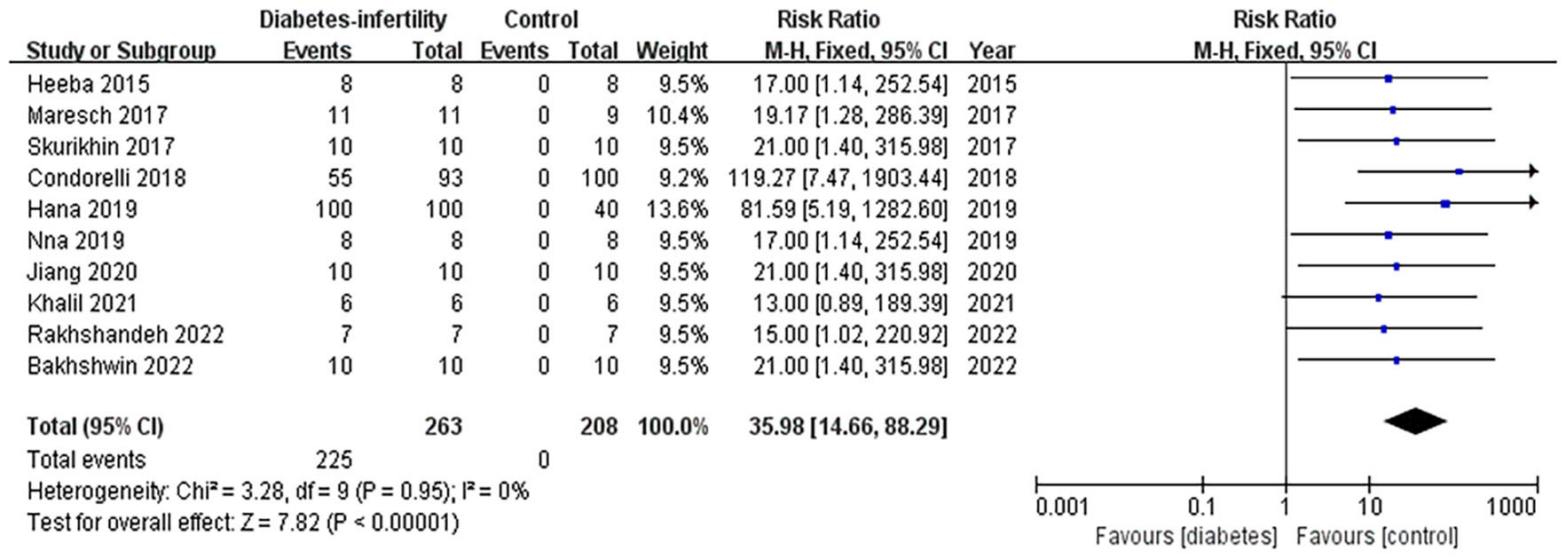
Meta-analysis of inflammation in diabetes-induced male infertility.

According to the forest plot (**Figure 5**), the *Z*-test for the overall effect showed significant statistics (*Z* = 7.82, *p <* 0.00001), indicating that inflammation is an important factor in diabetes-induced male infertility. Ten studies were included in the meta-analysis, involving 263 diabetes-induced infertility individuals, of whom 225 events showed increased inflammatory activity (RR = 35.98; 95% CI = 14.66, 88.29). The fixed-effects model revealed exceptional consistency across studies (*I-*square = 0%), eliminating concerns regarding interstudy heterogeneity (**Supplementary Figure S2**). This robust association underscores inflammation as a pivotal mediator in diabetes-associated male infertility.

### 3.3 Diabetes leads to male infertility through an inflammatory process

We searched two RNA-seq databases from the testes of diabetic male mice available at the NCBI related to diabetes-associated male infertility. Gene expression levels in the RNA sequencing data were estimated using the FPKM method. DEGs were screened using the criteria of *p* < 0.05 and fold change > 2.

**Figure 6:**
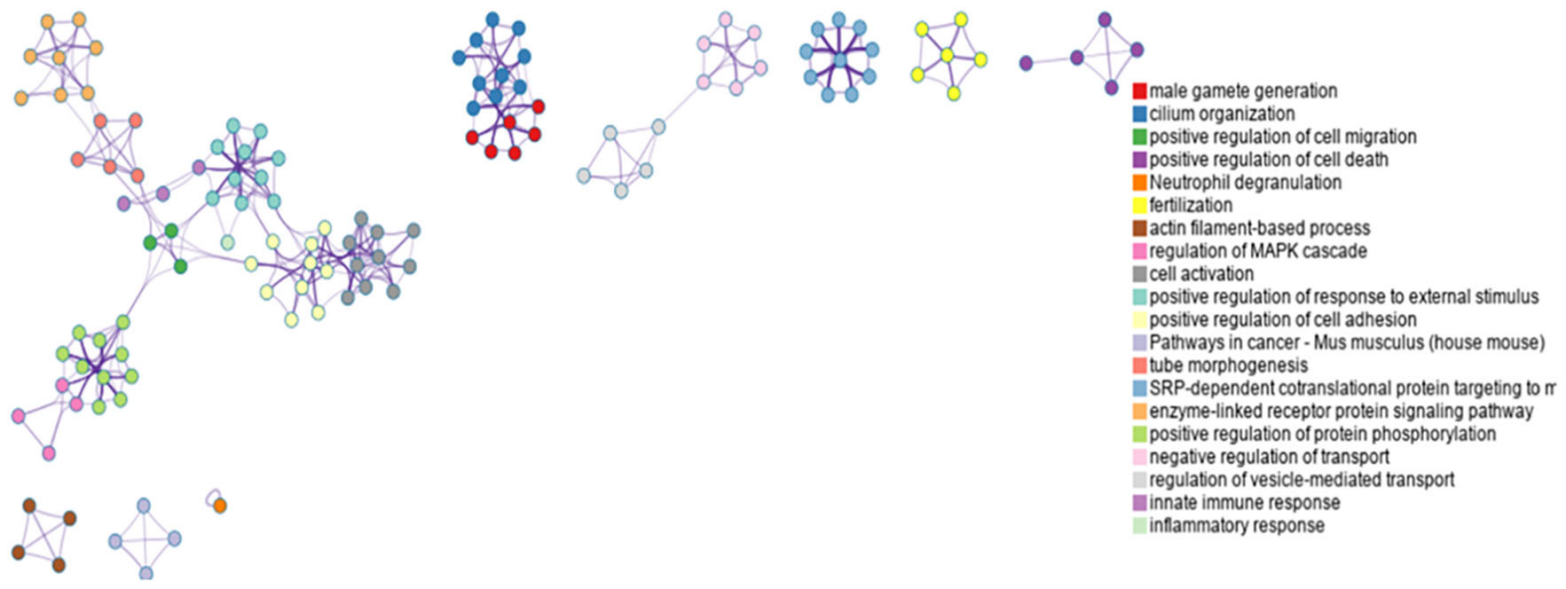
Genetic pathway enrichment analysis (https://metascape.org/).

Subsequently, the altered gene expression data from the diabetic and control groups were separated, combined, and further analyzed using an online analytical tool (http://metascape.org) (**Figure 2**). Based on the enriched pathways of the differentially expressed genes (**Figure 6**), most pathways were closely related to male fertility and included two notable pathways: innate immune response and inflammatory response. These findings suggest that diabetes can strongly influence male fertility through inflammatory pathways at the gene expression level.

**Figure 7:**
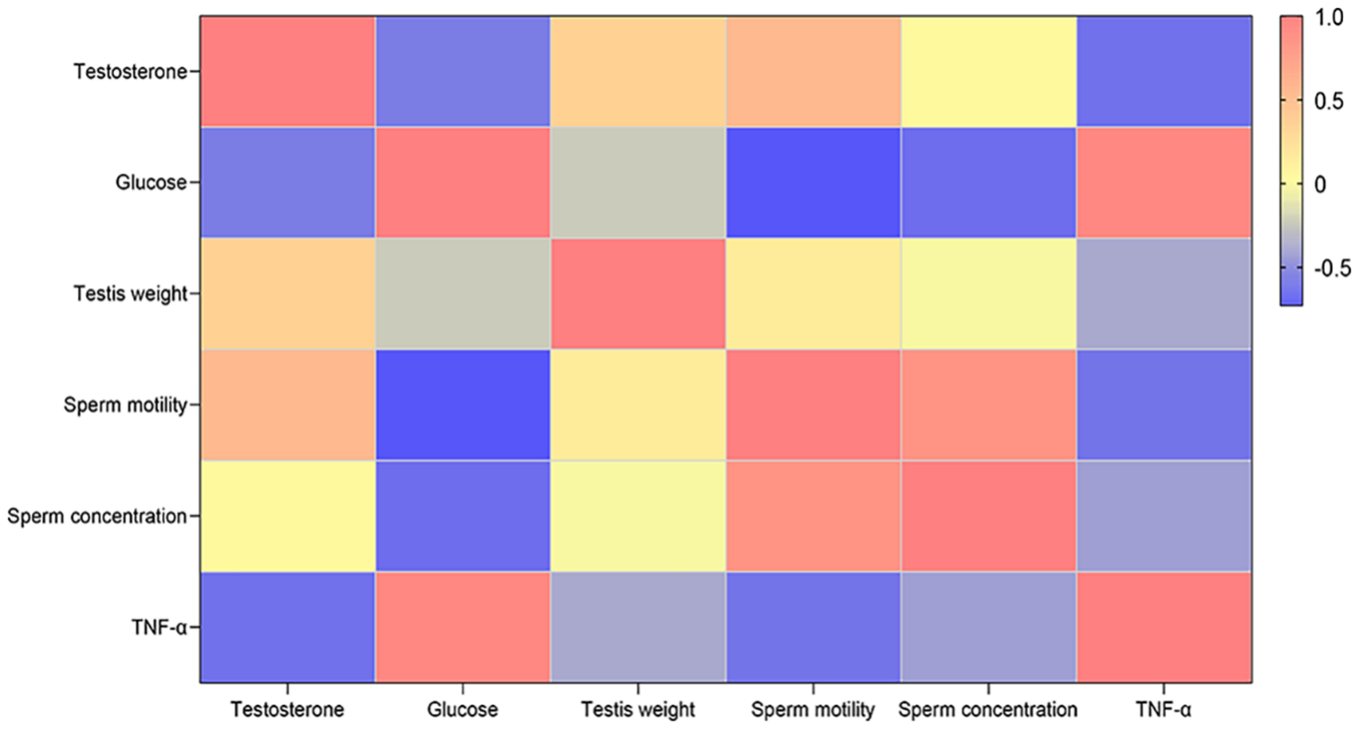
Correlation analysis of the TNF-α inflammatory pathway. Different colors represent different correlations. (Blue represents negative correlation; yellow represents no correlation; red represents positive correlation).

To explore interactions among diabetes, male infertility, and inflammation, we conducted a correlation analysis. The results demonstrated that the inflammatory pathway biomarker tumor necrosis factor alpha (TNF-α) was significantly negatively correlated with male fertility parameters, including testosterone, testis weight, sperm motility, and sperm concentration, particularly testosterone and sperm motility (**Figure 7**). In contrast, TNF-α was positively correlated with glucose levels. These findings provide further evidence of the role of inflammation in diabetes-induced male infertility. Diabetes disrupts the inflammatory system, contributing to male infertility.

## 4 Discussion

This is the first study to carry out a meta-analysis assessing the risk of male infertility in individuals with diabetes compared with controls. Based on the meta-analysis of the included studies, we propose that diabetes has a substantial impact on male reproduction. The underlying mechanisms are highly complex, and this paper primarily focuses on analyzing and discussing the role of inflammation.

Diabetes could lead to severe metabolic disease and complications (50). According to the IDF (71), diabetes rates are increasing worldwide. As early as 500–600 bc, two Indian physicians described the distinction between types 1 and 2 diabetes: type 1 being associated with onset in youth, and type 2 linked to obesity (51). Although types 1 and 2 diabetes show different characteristics, this study found that both have a substantial adverse effect on male fertility (3, 14, 26, 45, 52–55). This study included 27 papers for meta-analysis. Eight articles involving 422 individuals evaluated the risk of T1D on male fertility, yielding statistically significant results (*p* < 0.00001). Nine papers involving 850 individuals evaluated T2D in male fertility, also showing statistically significant results. Although some included studies had small sample sizes, the meta-analysis findings are consistent with our previous studies (56, 57). These results suggest that diabetes has a detrimental impact on male fertility. Interestingly, we found that type 2 diabetes was associated with a higher risk of male infertility than type 1 diabetes. This may be because type 2 diabetes is characterized by insulin resistance as its core feature and is accompanied by various complications, such as chronic hyperglycemia, lipid metabolism disorders (58), and systemic low-grade inflammation (59). These complications could negatively impact male reproductive function (26, 60).

Studies have established an association between inflammatory biomarkers and the occurrence of diabetes and its complications (61–63). According to our results, diabetes may induce male infertility through several pathways. Among them, inflammation (20.4%) was observed more frequently than hormonal (16.7%), apoptotic (13%), and oxidative (9.3%) pathways. The 10 studies included here involved 263 diabetes-induced male infertile individuals and 208 controls. The forest plot indicated that inflammatory reactions were active in diabetes-induced male infertility. These data consistently confirm that inflammatory pathways (TNF-α) serve as important mechanisms mediating male infertility in diabetes. Moreover, TNF-α showed a significant negative correlation with core male fertility indicators, such as sperm motility, sperm concentration, and testosterone levels, highlighting the causal role of inflammatory pathways in diabetes-induced male infertility and providing critical evidence for subsequent mechanistic research. Despite the strong consistency of the results, the inclusion of both animal and human studies introduced limitations and heterogeneity. A major limitation was the overreliance on rodent models in the included studies, which may reduce the applicability of the findings to human male fertility. This limitation primarily arises from significant differences between rodents and humans in physiological characteristics, metabolic mechanisms, and reproductive system structure. For instance, the spermatogenic cycle of mice (35 days) is much shorter than that of humans (74 days) (64, 65), and the ratio of Sertoli cells to germ cells in the seminiferous tubules also differs from that in humans (66). Such structural disparities may lead to variations in the duration and intensity of inflammatory effects on spermatogenesis. More importantly, the species-specificity of inflammatory pathways may limit the extrapolation of results. Here, we demonstrated that inflammation is a high-risk factor through which diabetes can cause male infertility. This analysis clarifies the mechanism by which diabetes induces male infertility via inflammatory pathways; however, the limitations of rodent models indicate that these findings require further validation in humans. In addition, genetic disorders were observed in diabetic male mice. Metascape analysis of gene expression in fertility pathways and inflammatory pathways further confirmed that inflammation plays a vital role in diabetes-induced male infertility. Pearson coefficient analysis showed the relationships among inflammation, diabetes, and male infertility. TNF-α is a cytokine with tumor necrosis activity and plays a role in inflammation (67). This study found that TNF-α is positively associated with diabetes and negatively associated with male fertility parameters, including testosterone, testis weight, sperm motility, and sperm concentration, confirming the results of the meta-analysis and gene expression pathway analysis. Another notable finding was that TNF-α was positively correlated with glucose levels. Studies have confirmed that elevated glucose is an important pathological feature of metabolic diseases such as type 2 diabetes and obesity (68). This association was not merely a concomitant phenomenon. The feedback regulation of TNF-α by glucose in the diabetes model creates a vicious cycle: high glucose levels stimulate the secretion of TNF-α, forming a vicious cycle of hyperglycemia→increased TNF-α→more severe hyperglycemia (69). Importantly, oxidative stress induced by high-sugar conditions negatively impacts sperm quality, which in turn indirectly affects normal male reproductive function (70). In addition, oxidative stress and hormonal imbalance are important contributors to male infertility in diabetes (54). The cycle of diabetes→oxidative stress→inflammation activation→hormonal imbalance→aggravated oxidative stress and inflammation→continuous damage to reproductive function→male infertility represents the core mechanism of male infertility in diabetes. Therefore, not only anti-inflammatory approaches, but also strategies targeting oxidative stress and hormonal imbalance, may be potential treatment directions for improving male infertility in diabetes. Consequently, comprehensive interventions addressing these three aspects—including anti-inflammatory therapy, antioxidant treatment, and testosterone supplementation—may become the key strategies for enhancing fertility in diabetic men in the future. Of course, this study represents a preliminary exploration in this field, and further high-quality studies are needed to validate these conclusions.

## 5 Conclusions

In summary, this meta-analysis demonstrates a significant risk of male infertility in diabetic populations, with T2D exhibiting greater reproductive toxicity than T1D. Mechanistically, chronic inflammation appears to be an important mediator, as indicated by correlation analysis and transcriptomic signatures. In the future, targeting inflammation may become a potential therapeutic strategy for diabetic male infertility.

## Data Availability

The datasets presented in this study can be found in online repositories. The names of the repository/repositories and accession number(s) can be found in the article/**Supplementary Material**.

## Supplementary material

Supplementary Figure 1 The funnel plot corresponding to **Figure 3**.

Supplementary Figure 2 The funnel plot corresponding to **Figure 5**.
