# Correction: Inflammation-mediated effects of diabetes mellitus on male fertility: a systematic review and meta-analysis

**DOI:** 10.3389/fendo.2025.1761350

**Published:** 2025-12-22

**Authors:** Yanan Hao, Zheng Yang, Yanni Feng, Yong Zhao, Yonglin Ren

**Affiliations:** 1 The Institute of Brain Science and Brain-inspired Research, Shandong First Medical University and Shandong Academy of Medical Sciences, Jinan, China; 2 College of Environmental and Life Sciences, Murdoch University, Perth, WA, Australia; 3 College of Pharmacy, Shandong First Medical University, Jinan, China; 4 College of Life Sciences, Qingdao Agricultural University, Qingdao, China; 5 Chinese Academy of Agricultural Sciences, Beijing, China

**Keywords:** type 1 diabetes, type 2 diabetes, inflammation, male infertility, meta-analysis

Affiliation “^3^College of Pharmacy, Shandong First Medical University” was omitted for author “Zheng Yang”. This affiliation has now been added for author “Zheng Yang”.

Author “Zheng Yang” was erroneously assigned to affiliation “^2^College of Environmental and Life Sciences, Murdoch University, Perth, WA, Australia”. This affiliation has now been removed for author “Zheng Yang”.

The original version of this article has been updated.

